# Effect of a fixed-dose combination of Telmisartan/S-amlodipine on circadian blood pressure compared with Telmisartan monotherapy: TENUVA-BP study

**DOI:** 10.1186/s40885-021-00184-0

**Published:** 2022-03-01

**Authors:** Bong-Joon Kim, Kyoung-Im Cho, Hyuck Moon Kwon, Seung-Min Choi, Chang-Hwan Yoon, Sang Wook Lim, Seung-Jae Joo, Nam Ho Lee, Sang-Yup Lim, Seong-Hoon Lim, Hyo-Soo Kim

**Affiliations:** 1 Division of Cardiology, Department of Internal Medicine, Kosin University College of Medicine, Gospel Hospital, Busan, Republic of Korea; 2 Cardiology Division, Department of Internal Medicine, Gangnam Severance Hospital, Yonsei University, Seoul, Republic of Korea; 3 Department of Cardiology, National Medical Center, Seoul, Republic of Korea; 4 Division of Cardiology, Department of Internal Medicine, Seoul National University Bundang Hospital, Seongnam, Republic of Korea; 5 Division of Cardiology, Department of Internal Medicine, CHA Bundang Medical Center, CHA University, Seongnam, Republic of Korea; 6 Department of Internal Medicine, Jeju National University Hospital, Jeju, Republic of Korea; 7 Department of Internal Medicine, Hallym University Kangnam Sacred Heart Hospital, Seoul, Republic of Korea; 8 Department of Cardiology, Korea University Ansan Hospital, Ansan, Republic of Korea; 9 Cardiovascular Division, Department of Internal Medicine, Dankook University Hospital, Cheonan, Republic of Korea; 10 Department of Internal Medicine, Seoul National University College of Medicine, Seoul, Republic of Korea

**Keywords:** Amlodipine, Circadian rhythm, Drug combinations, Essential hypertension, Telmisartan

## Abstract

**Background:**

This study evaluated the circadian efficacy of a telmisartan 40 mg/S-amlodipine 2.5 mg fixed-dose combination (Telmisartan40/S-Amlodipine2.5) compared to telmisartan 80 mg (Telmisartan80) in patients with essential hypertension who did not respond to 2–4 weeks’ treatment with telmisartan 40 mg.

**Methods:**

Eligible patients with essential hypertension (clinic mean sitting systolic blood pressure [MSSBP] ≥140 mmHg, or ≥ 130 mmHg in those with diabetes mellitus or chronic kidney disease) were randomly assigned to Telmisartan40/S-Amlodipine2.5 or Telmisartan80 for 8 weeks. All patients underwent ambulatory BP monitoring (ABPM) at baseline and 8 weeks later. Primary endpoints were changes in mean 24-h SBP and DBP on 24-h ABPM from baseline after 8 weeks. Secondary endpoints were changes in daytime, nighttime, and morning SBP and DBP, and clinic MSSBP and MSDBP.

**Results:**

A total of 316 Korean patients were enrolled, 217 patients were randomized to treatment, and 192 patients completed the study. Compared to Telmisartan80, Telmisartan40/S-Amlodipine2.5 showed significantly better reductions in 24-h mean SBP and DBP after 8 weeks. Telmisartan40/S-Amlodipine2.5 also significantly reduced secondary endpoints compared to Telmisartan80. Among 15 adverse events (7 [Telmisartan40/S-Amlodipine2.5] and 8 [Telmisartan80]), there were five adverse drug reactions; 14 events were mild, and none were identified with significant between-group differences.

**Conclusions:**

Telmisartan40/S-Amlodipine2.5 was tolerable and more effective than Telmisartan80 in lowering 24-h mean ambulatory BP in patients with essential hypertension not responding adequately to Telmisartan40. Our findings support the fact that the combination of S-amlodipine with telmisartan is more appropriate than increasing the dose of telmisartan monotherapy.

**Trial registration:**

ClinicalTrials.gov, NCT02231788. Registered 4 September 2014.

**Supplementary Information:**

The online version contains supplementary material available at 10.1186/s40885-021-00184-0.

## Background

According to the Korea Hypertension Fact Sheet 2020, with the rapid aging of the population, the absolute number of people with hypertension (HTN) has steadily increased; as of 2018, the number has exceeded 12 million. Since 1998, the HTN awareness rate has increased and treatment rates have improved; however, the control rate was 47%, a level that still needs further improvement [1]. In terms of recognition of the requirement for more active blood pressure (BP) control, the American Heart Association revised the diagnostic guidelines for HTN, reducing the cut-off to 130/80 mmHg. More than two-thirds of patients with hypertension require treatment with two or more antihypertensive drugs to achieve their target BP goals [2, 3]. The European Society of Cardiology recommends initial combination therapy for the majority of patients with HTN [4].

In the diagnosis and treatment of HTN, accurate measurement of BP is important in clinical practice, and a single BP measurement at an office is generally not adequate to assess BP fluctuations. According to the Korean ambulatory BP monitoring (ABPM) Registry for Evaluation of the Prognostic Threshold in Hypertension (Kor-ABP), about 30% of subjects not using ABPM were misdiagnosed in clinical practice regardless of their antihypertensive medication status [5]. Nighttime BP is also a stronger risk factor for coronary heart disease (CHD) and stroke than clinic BP, and a non-dipping pattern has been associated with an increased risk for cardiovascular (CV) events and all-cause mortality [6, 7]. Because ABPM can provide better prognostic information than BP measurements in the clinic, it is emphasized in guidelines, including those from the Korean Society of Hypertension.

It is well known that combination therapies are more effective than monotherapy in meta-analysis [8], and a renin-angiotensin system inhibitor + calcium channel blockers are the most commonly recommended combinations [9–11]. In particular, the combination of telmisartan and amlodipine has considerable clinical evidence supporting its beneficial antihypertensive efficacy for the longest half-life in each class [12, 13]. S-amlodipine is the more active isomer of amlodipine besylate, which is effective for HTN caused by fluid retention due to its additional natriuretic activity [14]. Telmisartan, which is long-acting and has a plasma half-life of 24 h [15, 16], acts selectively on angiotensin II receptors. Recently, a fixed-dose combination of S-amlodipine besylate 2.5 mg and telmisartan 40 mg has been developed for treating HTN.

There is a lack of studies that compare the effects of single drug and combination therapy on BP reduction, including circadian BP in patients with HTN but not resistant HTN. The aim of this study was to determine the circadian efficacy of combined HTN treatment with telmisartan 40 mg and S-amlodipine besylate 2.5 mg (Telmisartan40/S-Amlodipine2.5) compared with telmisartan 80 mg (Telmisartan80) in patients with HTN who did not respond to telmisartan 40 mg (Telmisartan40) monotherapy.

## Methods

### Study design

This was a multicenter, randomized, parallel-group comparative phase IV clinical trial followed by an 8-week open-label extension period (NCT02231788). Patients who agreed to participate in the clinical trial and met the inclusion and exclusion criteria had an initial telmisartan 40 mg treatment period of 2–4 weeks. Patients considered appropriate for this trial were randomly assigned at a 1:1 ratio to the Telmisartan40/S-Amlodipine2.5 group or to the control Telmisartan80 group. To ensure a balanced allocation of subjects, the randomization sequence generated using SAS version 9.4 (SAS Institute Inc., Cary, NC, USA) was used to construct a random block of 4, stratified by the participating center and the presence of diabetes mellitus (DM) and chronic kidney disease (CKD). All patients received Telmisartan40/S-Amlodipine2.5 or Telmisartan80 once daily during the 8-week treatment period (Fig. S1). The study protocol was approved by the Institutional Review Board at each institution. All eligible patients provided written informed consent to participate.

### Study populations

Patients were enrolled between May 2014 and March 2018. Inclusion criteria included age ≥ 19 years and clinic mean seated SBP (MSSBP) ≥140 mmHg (≥130 mmHg in patients with DM or CKD) despite treatment with telmisartan 40 mg during the initial treatment period. Exclusion criteria were extensive and included a clinic MSSBP ≥200 mmHg or a clinic mean seated DBP (MSDBP) ≥120 mmHg on screening and randomization; nighttime workers; patients with abnormal liver function (AST/ALT > 3 times the upper limit of normal [ULN]) or abnormal renal function (serum creatinine > 4 times ULN); and patients with secondary HTN (coarctation of aorta, Cushing’s syndrome, pheochromocytoma, and primary aldosteronism) except for HTN due to DM and CKD. Those with heart failure (New York Heart Association functional class III-IV); a history of myocardial infarction, unstable angina, significant valvular heart disease, or cardiac arrhythmia requiring treatment within the previous 3 months; a history of severe cerebrovascular disease such as a stroke or cerebral hemorrhage within the previous 6 months; and patients scheduled for renal transplantation during the clinical trial were also excluded. Other exclusion criteria included patients with severe or malignant retinopathy, patients with acute or chronic inflammation requiring treatment, a history of malignancy within 5 years, and a history of angioedema for angiotensin-converting enzyme inhibitor or angiotensin receptor blocker. Patients with known severe hypersensitivity to amlodipine or telmisartan; patients with a condition that may significantly affect the absorption, distribution, and excretion of the clinical trial drugs; patients in need of concomitant use of an HTN drug other than the clinical trial drugs during the period of the clinical trial; and patients who took other clinical trial drugs within the previous 30 days were similarly excluded. A history of alcohol or drug dependency, current pregnancy or lactation, and potential pregnancy without proper contraception also resulted in exclusion.

### Blood pressure measurement

All BP measurements were taken with sponsor-supplied sphygmomanometers (OMRON HEM-7080 IT from Omron Healthcare Co., Ltd. for clinic BP measurements, and Watch BP O3/3MZ1 from Microlife Corp. for ABPM measurements, NeiHu, Taipei). At visit 1, clinic BP was calculated as the mean of three measurements taken at 2-min intervals on both arms. The arm showing the higher SBP was used as the baseline, and that arm was used at future clinic visits. ABPM was performed on the right arm in the left-handed subjects and on the left arm in the right-handed subjects. All ABPM measurement were proceeded by 24 h clock-based definition.

### Study efficacy endpoints

The primary endpoints were the changes in the mean 24-h SBP and DBP on 24-h ABPM from the reference baseline after 8 weeks of the treatment period.

The secondary endpoints were:

1) The reduction from the reference baseline in the mean daytime (06:00–21:59) SBP and DBP on 24-h ABPM after 8 weeks of the treatment period.

2) The reduction from the reference baseline in the mean nighttime (22:00–05:59) SBP and DBP on 24-h ABPM after 8 weeks of the treatment period.

3) The reduction from the reference baseline in the mean morning (06:00–11:59) SBP and DBP on 24-h ABPM after 8 weeks of the treatment period.

4) The reduction from the reference baseline in the clinic mean seated SBP and DBP after 8 weeks of the treatment period.

5) The mean ABPM control rate at trough after 8 weeks of the treatment period:

- mean 24-h SBP < 130 mmHg and mean 24-h DBP < 80 mmHg
- mean daytime (06:00–21:59) SBP < 135 mmHg and mean daytime DBP < 85 mmHg
- mean nighttime (22:00–05:59) SBP < 120 mmHg and mean daytime DBP < 70 mmHg
- clinic MSSBP < 140 mmHg and MSDBP < 90 mmHg (clinic MSSBP < 130 mmHg and MSDBP < 80 mmHg in patients with DM or CKD)

6) The mean ABPM response rate at trough after 8 weeks of the treatment period:

- mean 24-h SBP reduction ≥10 mmHg and mean 24-h DBP reduction ≥10 mmHg
- clinic MSSBP reduction ≥10 mmHg and clinic MSDBP reduction ≥10 mmHg

### Safety endpoint

Safety evaluation was used to evaluate the incidence of adverse events. These events were determined by laboratory tests and physical examinations.

### Statistical analysis

Statistical analyses were performed with SAS ver. 9.4. Data normality was tested using the Kolmogorov-Smirnov test. Values are expressed as means ± SD for numerical variables or as numbers of participants and their percentages for categorical variables. Student’s *t*-test was conducted to confirm that the mean change in ABPM from baseline after 8 weeks was superior in the test group compared to the control group. The analysis of categorical data such as control rate and response rate was performed using the chi-squared test or Fisher’s exact test. A two-tailed *P*-value less than 0.05 was considered statistically significant.

## Results

### Study population

**Fig. 1:**
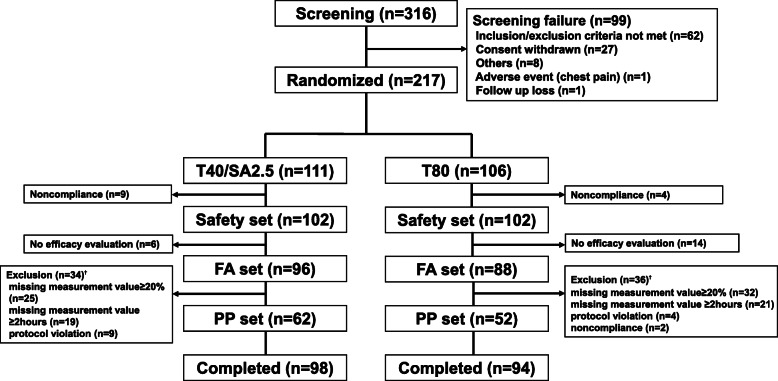
Patient disposition. T40/SA2.5, Telmisartan40/S-Amlodipine2.5; T80, Telmisartan80; FA, full analysis; PP, per protocol. Note: †Because there are duplicate subjects, the sum of each item is greater than the sum of the total

A total of 316 Korean patients were enrolled; of these, 99 patients were excluded (62 patients who did not meet the SBP criteria after treatment with telmisartan 40 mg during the initial treatment period, 27 patients who withdrew consent, and 10 others). Finally, 217 patients were randomized (*n* = 111 to the Telmisartan40/S-Amlodipine2.5 group and *n* = 106 to the Telmisartan80 group), and a total of 192 patients (*n* = 98 for Telmisartan40/S-Amlodipine2.5 and *n* = 94 for Telmisartan80) completed the final study protocol (Fig. 1). The mean age was 63.1 ± 11.7 years, and 57.1% were male. All patients had essential HTN; the mean duration of HTN was 11.6 ± 9.1 years. In baseline characteristics, there were no significant differences in age or sex. In addition, both groups showed similar prevalences of DM and CKD and similar durations of HTN (12.0 ± 9.5 vs. 11.2 ± 8.6 years, *P* = 0.580; Table 1). Of 184 subjects in the full analysis set, 89.1% had taken more than one drug before participating in the trial; there was no between-group difference (88.5% in Telmisartan40/S-Amlodipine2.5 vs 89.8% in Telmisartan80, *P* = 0.789). The mean compliance of the 184 subjects was 97.3% ± 5.3%, with no significant between-group difference (Telmisartan40/S-Amlodipine2.5 vs Telmisartan80, 97.5% ± 4.1% vs. 97.0% ± 6.3%, *P* = 0.505).

**Table 1:** Baseline characteristics

	All Subjects (*n* = 184)	Telmisartan40/S-Amlodipine2.5 (*n* = 96)	Telmisartan80 (*n* = 88)	*P*-value
Male (n, %)	105 (57.1)	59 (61.5)	46 (52.3)	0.209^a)^
Age, mean (year)	63.1 ± 11.7	63.5 ± 12.5	62.7 ± 10.9	0.650^b)^
Height (cm)	162.3 ± 8.6	163.0 ± 8.2	161.6 ± 9.0	0.270^a)^
Body weight (kg)	70.0 ± 13.8	69.6 ± 11.1	70.5 ± 16.3	0.650^a)^
HTN (n, %)	184 (100.0)	96 (100.0)	88 (100.0)	
Duration of HTN, mean (year)	11.6 ± 9.1	12.0 ± 9.5	11.2 ± 8.6	0.580^a)^
DM (n, %)	65 (35.3)	33 (34.4)	32 (36.4)	0.778^a)^
Duration of DM (year)	8.0 ± 9.2	7.3 ± 7.4	8.8 ± 10.8	0.532^b)^
CKD (n, %)	34 (18.5)	18 (18.8)	16 (18.2)	0.921^a)^
Office SBP (mmHg)	153.1 ± 15.5	154.8 ± 16.0	151.2 ± 14.9	0.111^a)^
Office DBP (mmHg)	89.2 ± 11.9	88.9 ± 12.5	89.6 ± 11.4	0.677^a)^
Heart rate (bpm)	73.2 ± 12.6	72.6 ± 13.3	73.9 ± 11.7	0.486^a)^
Body temperature (°C)	36.5 ± 0.2	36.5 ± 0.2	36.5 ± 0.2	0.920^a)^

^a)^Result of chi-square test. ^b)^ Result of Fisher’s exact for comparison between the two groups

HTN, hypertension; DM, diabetes mellitus; CKD, chronic kidney disease; SBP, systolic blood pressure DBP, diastolic blood pressure

### Primary endpoint

**Fig. 2:**
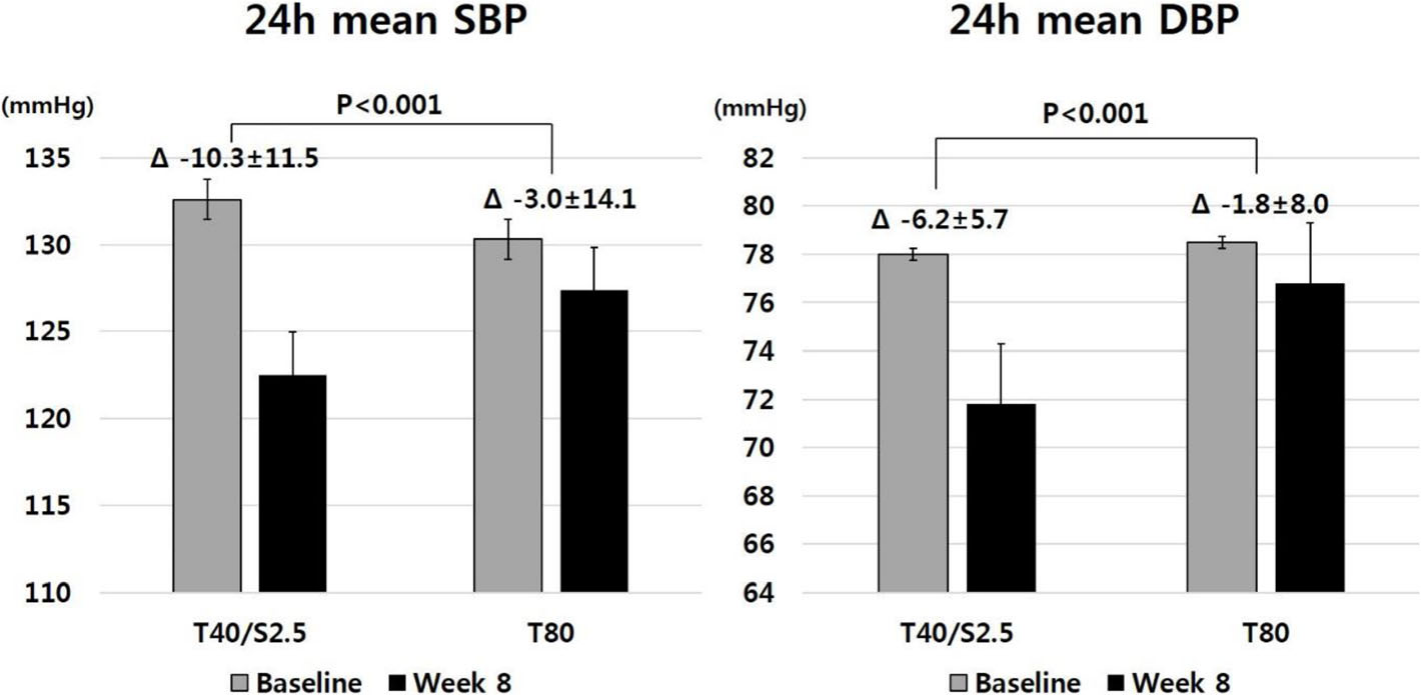
Comparison of changes in 24-h mean SBP/DBP according to treatment groups. SBP, systolic blood pressure; DBP, diastolic blood pressure; T40, Telmisartan40; SA2.5, S-Amlodipine2.5; T80, Telmisartan80

Subjects in the Telmisartan40/S-Amlodipine2.5 group showed a significantly greater reduction of 24-h mean SBP (− 10.3 ± 11.5 vs. − 3.0 ± 14.1 mmHg, *P* < 0.001) and DBP (− 6.2 ± 5.7 vs. − 1.8 ± 8.0 mmHg, *P* < 0.001) than those in the Telmisartan80 group after 8 weeks compared to baseline values (Table 2, Fig. 2). Compared to baseline, the mean DBP reduction was statistically significant in both groups (*P* < 0.001, *P* = 0.038), but the mean SBP reduction was significant only in the Telmisartan40/S-Amlodipine2.5 group (*P* < 0.001).

**Table 2:** Changes in 24-h ABPM: efficacy outcomes

	Telmisartan40/S-Amlodipine2.5 (*n* = 96)	Telmisartan80 (*n* = 88)	*P*-value^a)^
24 h mean SBP (mmHg)			
Baseline	132.6 ± 14.0	130.3 ± 13.8	
Week 8	122.5 ± 11.8	127.4 ± 15.6	
Change	−10.3 ± 11.5	−3.0 ± 14.1	< 0.001
24 h mean DBP (mmHg)			
Baseline	78.0 ± 10.6	78.5 ± 11.0	
Week 8	71.8 ± 8.1	76.8 ± 11.8	
Change	−6.2 ± 5.7	−1.8 ± 8.0	< 0.001
Nighttime mean SBP (mmHg)			
Baseline	128.5 ± 15.9	123.1 ± 14.7	
Week 8	115.4 ± 11.2	120.7 ± 18.0	
Change	−10.1 ± 14.7	−2.8 ± 17.1	0.003
Nighttime mean DBP (mmHg)			
Baseline	72.7 ± 10.8	73.3 ± 11.0	
Week 8	67.4 ± 8.1	72.0 ± 13.2	
Change	−5.2 ± 7.8	−1.4 ± 11.3	0.010
Mean daytime (06:00 ~ 21:59) SBP (mmHg)			
Baseline	135.3 ± 14.7	132.8 ± 14.4	
Week 8	125.1 ± 13.1	130.2 ± 15.6	
Change	−10.5 ± 11.9	−2.6 ± 14.2	< 0.001
Mean daytime DBP (06:00 ~ 21:59) (mmHg)			
Baseline	79.9 ± 11.1	80.3 ± 11.7	
Week 8	73.4 ± 8.8	78.8 ± 11.9	
Change	−6.6 ± 6.0	−1.6 ± 8.0	< 0.001
Mean morning-time (06:00 ~ 11:59) SBP (mmHg)			
Baseline	134.7 ± 13.6	133.6 ± 14.4	
Week 8	126.7 ± 14.1	130.7 ± 15.1	
Change	−8.4 ± 12.8	−3.1 ± 14.2	0.009
Mean morning-time (06:00 ~ 11:59) DBP (mmHg)			
Baseline	80.6 ± 9.9	81.0 ± 11.1	
Week 8	74.7 ± 9.4	79.2 ± 11.5	
Change	−6.0 ± 7.7	−1.8 ± 8.7	< 0.001
Mean office SBP (mmHg)			
Baseline	154.8 ± 16.0	151.2 ± 14.9	
Week 8	139.4 ± 16.7	145.3 ± 16.0	
Change	−15.4 ± 15.3	−5.9 ± 14.1	< 0.001
Mean office DBP (mmHg)			
Baseline	88.9 ± 12.5	89.6 ± 11.4	
Week 8	81.2 ± 11.13	88.0 ± 1.8	
Change	−7.7 ± 9.1	−1.6 ± 8.9	< 0.001

*ABPM* ambulatory blood pressure monitoring, *SBP* systolic blood pressure, *DBP* diastolic blood pressure

^a)^Result of independent t-test for comparison between two groups

### Secondary endpoints

The Telmisartan40/S-Amlodipine2.5 group showed significantly greater reductions in mean nighttime SBP (− 10.1 ± 14.7 vs. − 2.8 ± 17.1 mmHg, *P* = 0.003) and DBP (− 5.2 ± 7.8 vs. − 1.4 ± 11.3 mmHg, *P* = 0.010) (Table 2). The Telmisartan40/S-Amlodipine2.5 group also showed significantly lower reductions of mean daytime (06:00–21:59) SBP (− 10.5 ± 11.9 vs. − 2.6 ± 14.2 mmHg, *P* < 0.001) and DBP (− 6.6 ± 6.0 vs. − 1.6 ± 8.0 mmHg, *P* < 0.001), mean morning (06:00–11:59) SBP (− 8.4 ± 12.8 vs. − 3.1 ± 14.2 mmHg, *P* = 0.009) and DBP (− 6.0 ± 7.7 vs. − 1.8 ± 8.7 mmHg, *P* < 0.001) and clinic MSSBP (− 15.4 ± 15.3 vs. − 5.9 ± 14.1 mmHg, *P* < 0.001) and MSDBP (− 7.7 ± 9.1 vs. − 1.6 ± 8.9 mmHg, *P* < 0.001) compared to the Telmisartan80 group after 8 weeks compared to baseline values.

Table 3 shows the control rates and response rates of patients after 8 weeks. The percentages of patients who achieved 24-h-mean SBP/DBP < 130/80 mmHg in 8 weeks were 70.8% with Telmisartan40/S-Amlodipine2.5 and 45.5% with Telmisartan80. The percentages of patients who achieved daytime mean SBP/DBP < 135/85 mmHg and nighttime mean SBP/DBP < 130/80 mmHg in 8 weeks were 77.1 and 50.0%, respectively, with Telmisartan40/S-Amlodipine2.5 and 59.1 and 34.1%, respectively, with Telmisartan80. The control rate of office BP (< 140/90 mmHg) was 19.8% with Telmisartan40/S-Amlodipine2.5 and 9.1% with Telmisartan80. All values were significantly better with Telmisartan40/S-Amlodipine2.5 compared to Telmisartan80.

**Table 3:** Blood pressure control rate and response rate after 8 weeks

	Telmisartan40/S-Amlodipine2.5 (*n* = 96)	Telmisartan80 (*n* = 88)	*P*-value
BP control rate, n (%)			
Mean 24 h (00:00 ~ 23:59) SBP/DBP < 130/80 mmHg	68 (70.8)	40 (45.5)	< 0.001^a)^
Mean Daytime (06:00 ~ 21:59) SBP/DBP < 135/85 mmHg	74 (77.1)	52 (59.1)	0.009^a)^
Mean Nighttime (22:00 ~ 05:59) SBP/DBP < 130/80 mmHg	48 (50.0)	30 (34.1)	0.025^a)^
Clinic MSSBP/MSDBP < 140/90 mmHg	19 (19.8)	8 (9.1)	0.040^a)^
BP response rate, n (%)			
Mean 24 h (00:00 ~ 23:59) SBP/DBP reduction ≥10/10 mmHg	20 (20.8)	8 (9.1)	0.027^a)^
Clinic MSSBP/MSDBP reduction ≥10/10 mmHg	29 (30.2)	14 (15.9)	0.022^a)^

*BP* blood pressure, *SBP* systolic blood pressure, *DBP* diastolic blood pressure, *MS* mean sitting

^a)^Result of chi-square test

The percentages of patients who achieved 24-h-mean SBP/DBP reduction ≥10/10 mmHg after 8 weeks compared to baseline were 20.8% (20 of 96) in the Telmisartan40/S-Amlodipine2.5 group and 9.1% (8 of 88) in the Telmisartan80 group; there was a significant difference between the two groups (*P* = 0.027). The percentages of patients who achieved clinic MSSBP/MSDBP reduction ≥10/10 mmHg after 8 weeks compared to baseline were 30.2% (29 of 96) in the Telmisartan40/S-Amlodipine2.5 group and 15.9% (14 of 88) in the Telmisartan80 group; there was a significant between-group difference (*P* = 0.022).

When we analyzed the changes in BP variability in the 24-h ABPM data of our patients (Table S1), Telmisartan40/S-Amlodipine2.5 showed a tendency to reduce the standard deviation (SD) of 24-h SBP/DBP more, but there was no statistical significance (change of SD of 24-h SBP − 0.55 ± 4.26 vs. − 0.04 ± 3.87, *P* = 0.406, change of SD of 24-h DBP − 0.35 ± 2.94 vs. 0.14 ± 3.37, *P* = 0.296).

### Safety

Table 4 shows adverse events during the study period according to treatment group. Among the 204 subjects in the safety set, 13 patients (6.4%) experienced 15 cases of adverse events, and there was no difference between the two groups (6.9% in the Telmisartan40/S-Amlodipine2.5 group vs. 5.9% in the Telmisartan80 group, *P* = 0.774). Most (14 of 15) of the adverse events were mild; there were no serious adverse events. Adverse drug reactions (ADRs) were identified in 3 of the Telmisartan40/S-Amlodipine2.5 group patients (2.9%) and 2 of the Telmisartan80 group patients (2.0%); there was no between-group difference (*P* = 1.000). The three ADRs in the Telmisartan40/S-Amlodipine2.5 group were peripheral edema, increased LDL, and hypotension (1 case each). The two ADRs in the Telmisartan80 group were otitis media chronic (1 case) and generalized pruritus (1 case). All ADRs were also mild events; all were assessed as ‘recovering/resolving’ and ‘recovered/resolved’.

**Table 4:** Summary of adverse events during the study period according to treatment groups (Safety set)

	All Subjects (*n* = 204)	Telmisartan40/S-Amlodipine2.5 (*n* = 102)	Telmisartan80 (*n* = 102)	*P*-value
Overall AE, n (%) [no. of cases]	13 (6.37) [15]	7 (6.86) [7]	6 (5.88) [8]	0.774^a)^
ADR	5 (2.45) [5]	3 (2.94) [3]	2 (1.96) [2]	1.000^b)^
SAE	–	–	–	
**Infections and infestations**	3 (1.47) [3]	1 (0.98) [1]	2 (1.96) [2]	1.000^b)^
Herpes zoster	1 (0.49) [1]	1 (0.98%) [1]	–	1.000^b)^
Nasopharyngitis	1 (0.49) [1]	–	1 (0.98) [1]	1.000^b)^
Otitis media chronic	1 (0.49) [1]	–	1 (0.98) [1]	1.000^b)^
**Skin and subcutaneous tissue disorders**	3 (1.47) [3]	2 (1.96) [2]	1 (0.98) [1]	1.000^b)^
Photosensitivity reaction	1 (0.49) [1]	1 (0.98) [1]	–	1.000^b)^
Pruritus generalised	1 (0.49) [1]	–	1 (0.98) [1]	1.000^b)^
Urticaria	1 (0.49) [1]	1 (0.98) [1]	–	1.000^b)^
**Investigations**	2 (0.98) [2]	1 (0.98) [1]	1 (0.98) [1]	1.000^b)^
Blood glucose increased	1 (0.49) [1]	–	1 (0.98) [1]	1.000^b)^
Low density lipoprotein increased	1 (0.49) [1]	1 (0.98) [1]	–	1.000^b)^
**Nervous system disorders**	2 (0.98) [2]	–	2 (1.96) [2]	0.498^b)^
Headache	1 (0.49) [1]	–	1 (0.98) [1]	1.000^b)^
Migraine without aura	1 (0.49) [1]	–	1 (0.98) [1]	1.000^b)^
**Cardiac disorders**	1 (0.49) [1]	–	1 (0.98) [1]	1.000^b)^
Palpitations	1 (0.49) [1]	–	1 (0.98) [1]	1.000^b)^
**Eye disorders**	1 (0.49) [1]	–	1 (0.98) [1]	1.000^b)^
Dry eye	1 (0.49) [1]	–	1 (0.98) [1]	1.000^b)^
**General disorders and administration site conditions**	1 (0.49) [1]	1 (0.98) [1]	–	1.000^b)^
Oedema peripheral	1 (0.49) [1]	1 (0.98) [1]	–	1.000^b)^
**Psychiatric disorders**	1 (0.49) [1]	1 (0.98) [1]	–	1.000^b)^
Insomnia	1 (0.49) [1]	1 (0.98) [1]	–	1.000^b)^
**Vascular disorders**	1 (0.49) [1]	1 (0.98) [1]	–	1.000^b)^
Hypotension	1 (0.49) [1]	1 (0.98) [1]	–	1.000^b)^

^a)^Result of chi-square test. ^b)^ Result of Fisher’s exact for comparison between two groups

*AE* adverse event, *ADR* adverse drug reaction, *SAE* serious adverse event

## Discussion

Current Korean guidelines (The 2018 Korean Association of Hypertension Guidelines) suggest that if there is no adequate response to single drug therapy at stage 1 HTN with low to moderate risk, as an option in the next treatment plan, it may be possible to change to another class of drug, to increase the single drug dose, or to use combination therapy. Of course, it is difficult to apply the same guidelines because the biological and environmental characteristics of Westerners and Asians are different, but this is considered less aggressive than guidelines from Europe or the United States for combination treatment. In our study, the enrolled patients were relatively older, had a long duration of HTN, and did not respond to relatively low doses of HTN medication. In these patient groups, Telmisartan40/S-Amlodipine2.5 reduced 24-h mean SBP and DBP to a significantly greater extent than Telmisartan80. So, our results showed that even in Asian patients with ‘not severe’ HTN, the combination is more effective in lowering the BP, it supports the recent trend in which initial combination is emphasized. Several clinical trials have shown a greater antihypertensive effect with the amlodipine and telmisartan combination. Previous studies showed that the telmisartan/amlodipine combination was superior to amlodipine monotherapy in reducing BP (TEAMSTA-5 study) [13], and S-amlodipine/telmisartan exhibited superior antihypertensive effects compared with S-amlodipine monotherapy in the Korean population [17]. Compared to these studies, the significant feature of our study was that the diagnosis and therapeutic effects on circadian BP were all evaluated by 24-h ABPM. Clinic BP can be affected significantly by temperature, physical activity, emotional stress, caffeine, and alcohol. In particular, the white coat effect is a major cause of HTN misdiagnosis [18]. Therefore, due to the large number of measurements, ABPM provides a reliable estimate of an individual’s BP and can be used as an important tool to measure BP and to accurately determine the effects of HTN therapy [19], and it also provides information on BP during daytime activity and sleep, making ABPM a better predictor of target organ damage than clinic BP [20].

Another important finding of our study was that Telmisartan40/S-Amlodipine2.5 also significantly lowered nighttime, daytime, and morning BPs as well as 24-h mean BP compared to Telmisartan80. Nighttime HTN and morning HTN also have an important meaning and they are associated with a higher risk of total mortality and all CV events [21, 22]. Especially in the Asian population, nighttime HTN is more common due to high salt intake and sensitivity [23]. Although it is difficult to clearly explain this mechanism, it is possible that the synergistic effect of the telmisartan/amlodipine combination was more effective in correcting arteriolar vasodilatation and arterial stiffness [24–26]. This pathophysiology can be understood as the concept of BP variability. Although BP variability is not synonymous with BP reduction, in terms of effective BP control, BP variability is also clinically important and it is associated with CV events [27]. A previous study showed that the telmisartan/amlodipine combination was associated with a smoother BP reduction over 24 h and with a more favorable balance between mean 24-h BP reduction and the degree of BP variability on treatment, reflecting both its effectiveness in lowering BP levels and its longer duration of action [28]. Although our results have not statistically proven that Telmisartan40/S-Amlodipine2.5 lowers BP variability more than Telmisartan80, perhaps this is due to the insufficient number of enrolled patients. Therefore, even considering the long-acting characteristics of telmisartan, our result may support that Telmisartan40/S-Amlodipine2.5 is more effective to control BP constantly and stably than Telmisartan80.

During the study period, a total of 15 adverse events were identified, and most of them (14 of 15; 1 was considered moderate) were mild. There were also no severe ADRs. Our safety findings align with those from other studies using telmisartan [29]. The incidence of adverse events suggests that the Telmisartan40/S-Amlodipine2.5 combination is relatively tolerable. Peripheral edema was noted in one patient in the Telmisartan40/S-Amlodipine2.5 group (1.0%), a finding that is usually associated with amlodipine; the incidence of edema with conventional amlodipine therapy is ≥10% [30]. A previous study showed that with S-amlodipine, the incidence of peripheral edema was lower [30], whereas another study also showed that S-amlodipine was associated with less ankle edema than amlodipine besylate in Korean women with mild to moderate HTN [31]. This may be due to the S-amlodipine dosage being equivalent to half the dosage of amlodipine besylate. In addition, S-amlodipine is effective against HTN caused by fluid retention (renin-independent) by acting on sodium diuretics as well as influencing vasodilation [14, 32]. Conventional amlodipine also releases nitric oxide through stimulation of inducible nitric oxide synthase in a concentration-dependent manner, whereas S-amlodipine does not. This may be a reason for the lower incidence of peripheral edema [33]. Our findings support the fact that Telmisartan40/S-Amlodipine2.5 is tolerable compared to Telmisartan80 in terms of peripheral edema.

Our study has several limitations. First, this is an unblinded trial. Second, the number of enrolled patients was small, and the study duration was short. Therefore, our study is limited to short-term BP reduction effects, and there are limitations in evaluating long-term BP lowering effects or CV outcomes. Third, our study did not strictly control for lifestyle modification. Fourth, the study population was all Korean, making generalizations to other ethnic groups difficult. However, our study has the strength of measuring BP by ABPM at diagnosis and follow-up, allowing us to minimize errors of BP measurement and evaluate circadian BP variation.

## Conclusions

Compared to Telmisartan80, the Telmisartan40/S-Amlodipine2.5 combination was more effective in lowering circadian BP in patients with essential HTN who did not respond adequately to Telmisartan40. There was no difference in safety. Our findings support the hypothesis that combining S-amlodipine with telmisartan is more appropriate than increasing the dose of telmisartan monotherapy.

## Supplementary Information

**Additional file 1: Study design**

**Additional file 2: Changes in BP variabilityafter treatment**

## Data Availability

The datasets used and/or analysed during the current study are available from the corresponding author on reasonable request.

## Abbreviations

ABPM: Ambulatory blood pressure monitoring
ADR: Adverse drug reaction
BP: Blood pressure
CKD: Chronic kidney disease
CV: Cardiovascular
CVD: Cardiovascular disease
DBP: Diastolic blood pressure
DM: Diabetes mellitus
HTN: Hypertension
MS: Mean sitting
MSDBP: Mean sitting diastolic blood pressure
MSSBP: Mean sitting systolic blood pressure
SBP: Systolic blood pressure
SD: Standard deviation
ULN: Upper limit of normal
