# Supplementary material for: Effect of a fixed-dose combination of Telmisartan/S-amlodipine on circadian blood pressure compared with Telmisartan monotherapy: TENUVA-BP study
Source: Clin Hypertens. 2022 Mar 1;28:7. doi: 10.1186/s40885-021-00184-0 (PMC8886760; doi:10.1186/s40885-021-00184-0)
Supplement: Supplementary file 1 — Additional file 1: Study design [file 40885_2021_184_MOESM1_ESM.docx]

**Supplementary File – Fig. S1**

Study design

|  | | **[Treatment Period]** |  |
| --- | --- | --- | --- |
|  | **[Run-in Period]** | Telmisartan40/S-Amlodipine2.5 |  |
| Telmisartan 40mg |  |  |  |
|  |  |  |  |
|  |  | Telmisartan80 |  |
|  | 0W  [Visit 2]  D0+5  -4~-2W  [Visit 1]  D-33~D-14 | 8W  [Visit 3]  D56±5 |  |

Patients had an initial telmisartan 40 mg treatment period of 2–4 weeks. Patients were randomly assigned at a 1:1 ratio to the Telmisartan40/S-Amlodipine2.5 group or to the control Telmisartan80 group. All patients received Telmisartan40/S-Amlodipine2.5 or Telmisartan80 once daily during the 8-week treatment period.

Telmisartan40/S-Amlodipine2.5 = telmisartan 40 mg/S-amlodipine 2.5 mg, Telmisartan80 = telmisartan 80 mg.
