# Supplementary material for: Effect of a fixed-dose combination of Telmisartan/S-amlodipine on circadian blood pressure compared with Telmisartan monotherapy: TENUVA-BP study
Source: Clin Hypertens. 2022 Mar 1;28:7. doi: 10.1186/s40885-021-00184-0 (PMC8886760; doi:10.1186/s40885-021-00184-0)
Supplement: Supplementary file 2 — Additional file 2: Changes in BP variabilityafter treatment [file 40885_2021_184_MOESM2_ESM.docx]

**Supplementary File - Table S1**

Changes in BP variability after treatment

|  | Telmisartan40/S-Amlodipine2.5  (n = 96) | Telmisartan80  (n = 88) | P-value^a)^ |
| --- | --- | --- | --- |
| SD of 24h (00:00~24:00) SBP (mmHg) |  |  |  |
| Baseline | 14.8 ± 4.4 | 15.1 ± 4.0 |  |
| Week 8 | 14.3 ± 4.0 | 15.0 ± 3.6 |  |
| Change | −0.55 ± 4.26 | −0.04 ± 3.87 | 0.406^a)^ |
| SD of 24h (00:00~24:00) DBP (mmHg) |  |  |  |
| Baseline | 10.1 ± 2.9 | 10.4 ± 3.3 |  |
| Week 8 | 9.7 ± 2.8 | 10.5 ± 2.6 |  |
| Change | −0.35 ± 2.94 | 0.14 ± 3.37 | 0.296^a)^ |

SD, standard deviation; SBP, systolic blood pressure; DBP, diastolic blood pressure.

^a)^Result of chi-square test.
